# Randomized Trial of Adding Parenteral Acetaminophen to Prochlorperazine and Diphenhydramine to Treat Headache in the Emergency Department

**DOI:** 10.5811/westjem.2016.12.29218

**Published:** 2017-02-27

**Authors:** Stefan H. Meyering, Ryan W. Stringer, Matthew K. Hysell

**Affiliations:** Michigan State University, Lakeland Healthcare, Department of Emergency Medicine, St. Joseph, Michigan

## Abstract

**Introduction:**

Headaches represent over three million emergency department (ED) visits per year, comprising 2.4% of all ED visits. There are many proposed methods and clinical guidelines of treating acute headache presentations. However, data on intravenous acetaminophen usage in these settings are lacking. In this study, we sought to determine the efficacy of intravenous (IV) acetaminophen as an adjunct to a standard therapy for the treatment of patients who present to the ED with a chief complaint of “headache.”

**Methods:**

We conducted a single site, randomized, double-blind, placebo-controlled trial investigating the clinical efficacy of IV acetaminophen as an adjunct to a standard therapy with prochlorperazine and diphenhydramine for the treatment of patients who present to the ED with a chief complaint of “headache” or variants thereof. (See below for variants). The primary outcome measure of the efficacy of parenteral acetaminophen as an adjunct treatment for headache in addition to a standard therapy was a threshold two-point reduction in visual analog scale (VAS) pain scores on a 1–10 level at 90 minutes. Secondary outcomes measures included assessment of decreased requirement of “rescue” pain medicines, defined as any analgesic medications outside of diphenhydramine, prochlorperazine and acetaminophen, with particular interest to potential opioid-sparing effects with parenteral acetaminophen. Additional secondary outcome measure included time to disposition from arrival in the ED.

**Results:**

For the acetaminophen group the initial mean pain score was 8.67, for the placebo group 8.61. At 90 minutes pain score was 2.23 for the acetaminophen group and 3.99 for placebo (p<0.01, 95% confidence interval (CI) [0.8%–16%]. Of 45 patients in each group, we observed at least a threshold two-point decrease in pain score 36/45 (80%) with acetaminophen vs. 25/45 (55%) with placebo (p <0.01) 95% CI [5%–41%], number needed to treat (NNT) = 4). Secondary outcome measure did not demonstrate a difference in length of stay (161 minutes for acetaminophen arm and 159 minutes for placebo). However, 17/45 (38%) of patients who received IV acetaminophen required rescue analgesia, opposed to 24/45 (53%) of patients in the placebo group (p=0.13) 95% CI [−5%–34%].

**Conclusion:**

IV acetaminophen when used with prochlorperazine and diphenhydramine to treat acute headaches in the ED resulted in statistically significant pain reduction compared with prochlorperazine and diphenhydramine alone as measured by both threshold of lowering VAS pain score by at least two points (NNT = 4) and overall decline in VAS pain score. Further study is required to validate these results.

## INTRODUCTION

Headaches represent over three million emergency department (ED) visits per year comprising 2.4% of all ED visits.^1^ Headache is among the three most common complaints of patient presentations to EDs across the country with 1,626 visits per 100,000 in the 18–44 age group.^1^ Hospitalization costs totaling over $408 million were reported as of 2008.^2^ Treatment of acute headache remains complex, often requiring an individualized regimen. There are many proposed methods and clinical guidelines of treating acute headache presentations; however, data on intravenous (IV) acetaminophen usage in these settings is lacking,^2^,^3^ IV acetaminophen had demonstrated success in the post-operative period found by retrospective medical use evaluation surveys at sparing opioids as a part of a multi-modal approach to analgesia.^4^. It has also displayed effectiveness in the treatment of acute renal colic when compared to morphine directly.^5^ While current recommendations for acute headache treatment do not routinely include opioids, many patients regularly use or require some form of opioid analgesia, complicating current approaches. Assessing the usage of IV acetaminophen in the setting of acute headache, as an adjunct to standard therapy and as part of a multi-modal approach, may display increased efficacy in terms of pain reduction and opioid-sparing effects.

## METHODS

We conducted a single site, randomized, double-blind, placebo-controlled trial investigating the clinical efficacy of IV acetaminophen as an adjunct to a standard therapy for the treatment of patients who present to the ED with a chief complaint of “headache” or variants thereof. (See below for variants.) Independent of the clinician’s ultimate disposition of the patient, data collection was performed to ascertain the primary outcome measure of the efficacy of parenteral acetaminophen as an adjunct treatment for headache in addition to a standard therapy, with primary end point being threshold two-point reduction in visual analog scale (VAS) pain scores on a 1–10 level at 90 minutes. An estimated 100 patients were needed for the study (50 patients in each group) to achieve adequate power when considering the primary outcome measure. The primary outcome measure would be anticipated to reflect a statistically significant difference in mean pain scores between acetaminophen and placebo greater or equal to two with standard statistical thresholds of p < 0.05 and beta (power) > 0.8. Secondary outcomes measures included assessment of decreased requirement of “rescue” pain medicines defined as any analgesic medications outside of the aforementioned protocol with particular interest to potential opioid-sparing effects with parenteral acetaminophen. Additional secondary outcome measures included decreased time to disposition from arrival in the ED.

We obtained institutional review board approval and informed consent documentation prior to beginning patient enrollment. We included a convenience sample of patients age 18–65 years presenting with chief complaint of headache, migraine headache, tension headache, cluster headache or headache not otherwise specified, reporting pain as >4 using a standard 10-point VAS. We excluded patients who consumed a cumulative dose of acetaminophen >2,600 mg within the preceding 24 hours (per manufacturer recommendations), physical or mental disability hindering adequate response to assessment of pain, mental disability limiting ability to give consent, hemodynamic instability or medical condition requiring acute lifesaving intervention, documented or suspected pregnancy or active breastfeeding, any known contraindication to acetaminophen use (liver failure, cirrhosis, hypersensitivity, allergic reactions), brain mass/glioma, intra-cranial hemorrhage, skull fracture and any contraindication or reported allergy to the use of prochlorperazine and/or diphenhydramine.

**Population Health Research Capsule**

What do we already know about this issue?

IV acetaminophen has showed promise in post-operative pain control trials with demonstrated narcotic-sparing effects. It has not yet shown much success for other pain presentations.

What was the research question?

Can the addition of IV acetaminophen to a “standard” headache cocktail help improve pain control, diminish length of stay, and decrease amount of “rescue’”medications?

What was the major finding of the study?

IV acetaminophen added to prochlorperazine and diphenhydramine to treat acute headaches in the ED resulted in significant pain reduction when compared with prochlorperazine and diphenhydramine alone, decreasing VAS pain scores by at least 2 points (NNT =4), with overall decline in VAS pain score.

How does this improve population health?

Non-narcotic treatment of acute pain in the ED is recommended. We evaluated the effectiveness of alternative regimen for a common complaint.

Patients presenting to the ED with chief complaint of headache or variant thereof were evaluated by the treating emergency physician, who discussed the study in detail with the patient, reviewed inclusion and exclusion criteria, and obtained informed consent for enrollment. An order set was used in the electronic medical record to initiate a pre-selected order cluster including prochlorperazine 10mg IV bolus, diphenhydramine 25mg IV bolus, 1,000 ml 0.9% normal saline bolus, and “study drug.” The “study drug” was either 100ml 0.9% sodium chloride in a minibag, or 1,000 mg IV acetaminophen transferred from the manufacturer’s vial into a 100 cc minibag, both labeled “study drug.” All patients received prochlorperazine, diphenhydramine, and 1,000 ml 0.9% normal saline immediately from the ED medication-dispensing machine, and then subsequently the “study drug” upon arrival from pharmacy via tube system to ensure blinding. Both IV acetaminophen and placebo were administered via IV infusion over a 15-minute interval as is required by the manufacturer’s dosing administration instructions. The study was double blinded to both physician and patient. Therefore, patients were randomized by the pharmacist to either treatment arm “A” or “B,” where “A” represented acetaminophen, and “B” represented placebo. The pharmacists used a numeric identifier in a logbook to track whether patients received the study drug or placebo.

ED nursing staff completed a stratification form that noted the patient age, chief complaint, pain assessment intervals at time of arrival, time of “study drug” administration, reassessment at 30-minute intervals thereafter, and additional reassessment if a “rescue” medication was later used. In the event of adverse reaction to the IV infusion of the “study drug,” the infusion would be stopped and pharmacy contacted if required to “break” the double blinding to determine which medication was administered.

One hundred patients were enrolled in the study from November 2014–June 2015. We excluded four enrolled patients from data analysis secondary to age; two were excluded for repeat enrollment (only the initial enrollment was included) and three were excluded secondary to missing data. One patient who was found to have a brain mass was also excluded.

## RESULTS

Forty-five patients received placebo and 45 IV acetaminophen. Both groups received 50 mg IV diphenhydramine, 10 mg IV prochlorperazine, and 1,000 ml 0.9% NS bolus. At no time was the study blinding broken secondary to patient side effects. Our patients’ racial demographics are reported in table.

**Figure 1:**
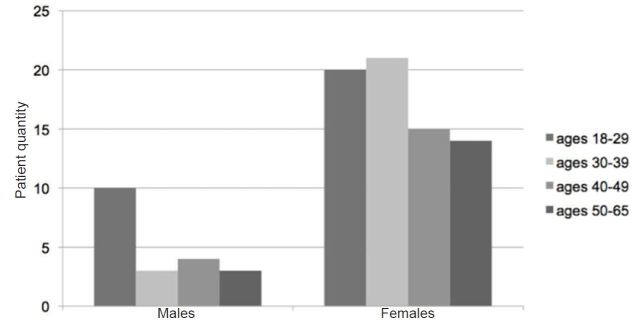
Demographics of patients in age range categories.

We enrolled 70 men and 20 women with a mean age of 31 and 38 respectively. Age groups of study participants were further divided with notable findings of the majority of males being within ages 18–29, and females being ages 18–39 (Figure 1).

**Figure 2:**
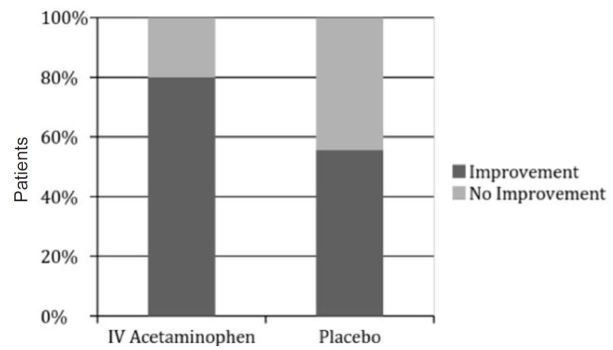
Improvement of visual analog scale pain score reporting ≥ 2 decrease from presentation at the 90-minute mark. *IV,* intravenous

Pain scores were analyzed at 0, 30, 60, and 90 minutes after study-drug administration. Pain scores were reported with ascending severity on a 1–10 point VAS. Of the (n=45) patients who received IV acetaminophen, 36 (80%) reported a decrease by pain score reporting of ≥ 2 from presentation at the 90-minute mark. Nine patients reported pain scores that were unchanged from initial presentation, increased, or decreased by <2 at the 90-minute assessment. Of the (n=45) patients who received placebo, 25 (55%) reported a decrease by pain score reporting of ≥ 2 from presentation at the 90- minute mark. Twenty patients reported pain scores that were either increased, unchanged from initial presentation, or decreased by <2 at the 90-minute assessment (p <0.01) 95% confidence interval [CI] (5%–41%) (Figure 2).

Forty-one patients required some form of “rescue” medication in addition to the initially administered medications; 17/45 (38%) of patients who received IV acetaminophen required rescue analgesia, as opposed to 24/45 (53%) of patients in the placebo group, which did not reach statistical significance.

**Figure 4:**
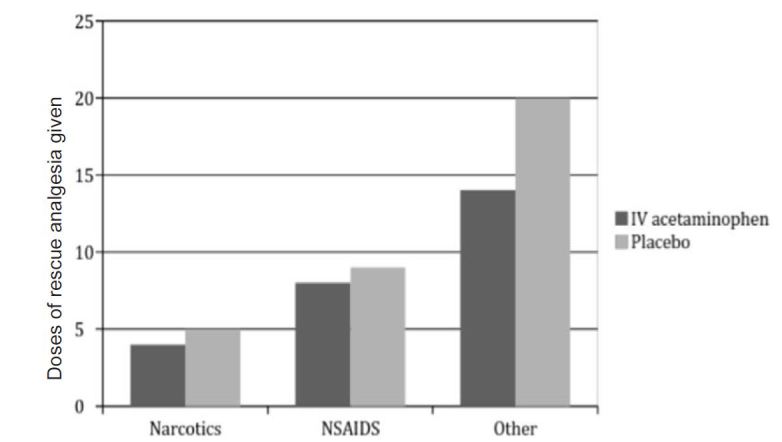
Numeric comparison of rescue analgesics for both intravenous acetaminophen and placebo arms. (p=0.13) 95% CI (−5%–34%) *NSAIDS,* non-steroidal anti-inflammatory drugs

Seventeen out of the 41 patients who required rescue analgesia received IV ketorolac as part of the rescue regimen: eight in the IV acetaminophen treatment arm and nine in the placebo arm. Nine patients received opioids as part of a rescue formulation: four in the IV acetaminophen treatment arm and five in the placebo arm (p=0.72). The opioids administered included hydrocodone, hydromorphone, meperidine, and fentanyl. Some patients received combination rescue medications including opioids and NSAIDS alone, in combination or in addition to other medications including orphenadrine, triptans, and steroids depending on clinician discretion (Figure 4). The level of “opioid-sparing effect” was not felt to be significant in this case, and was further confounded by co-administration of different classes of rescue medications.

**Figure 5:**
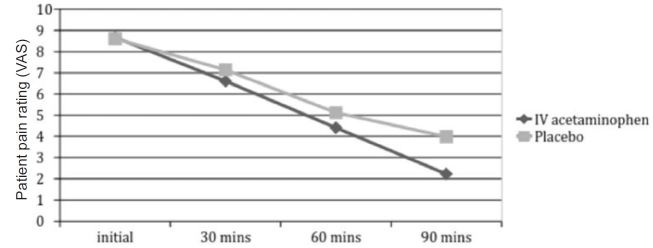
Distributions of the mean visual analog pain scores at predefined intervals from time of patient arrival in intravenous (IV) acetaminophen and placebo arms. (p=0.13), 95% CI [−5%–34%].

Mean time to clinically significant reduction in pain score as defined by at least a two-point decrease was 49.2 minutes post administration of IV acetaminophen, prochlorperazine and diphenhydramine. Mean time to clinically significant pain reduction was 71.3 minutes post administration of IV 0.9% NS, prochlorperazine and diphenhydramine. Mean pain intensity scoring (VAS) was noted for both groups. For the acetaminophen arm the initial mean pain score was 8.67, for the placebo arm 8.61. At 30, 60, and 90 minutes, corresponding mean pain scores were 6.61, 4.41, and 2.23 for the acetaminophen group, and 7.14, 5.12, 3.99 for the placebo group. A statistically significant difference in mean pain score was not observed until the 90-minute mark (p<0.01, CI 95% (0.8%–16%))(Figure 5).

Mean length of stay (LOS) was 161 minutes for the acetaminophen arm and 159 minutes for the placebo arm. LOS was extrapolated from the time of physician contact to disposition entered in the electronic health record (EHR) and included (in both groups) additional rescue medications and additional reassessment times. The maximum LOS for either treatment arm was 361 minutes. Disposition for two patients was admission and thus associated LOS was excluded from analysis. All other patients were discharged.

## DISCUSSION

Treatment of headaches in the clinical setting is difficult and requires an evidence-based and often patient-tailored approach, as there is a paucity of published data suggesting optimal headache therapy.^6^ The American Headache Society recommendations have endorsed certain medications as effective for various headache presentations including triptans, ergotamine derivatives, NSAIDs, opioids, and combination medications.^7^ As of late, there has been a significant driving force in the medical community to reduce the application of opioids.^2^,^3^ Opioids used routinely in headache presentations are not widely considered standard monotherapy, as they can contribute to rebound effects, increased reliance and addiction.^6^,^7^ Colman et. al. discovered a significantly increased likelihood of patient return to the ED within seven days with first-line opioid treatment of headache.^8^ Several adverse effects are associated with opioid use^3^ and may lead to prolonged recovery times, increased length of hospital stay, and higher incurred costs to the institution when applied to postoperative pain management strategies. Using multi-modal therapy with non-opioid agents is likely to be beneficial to both physicians and patients alike.^2^,^9^

Clinical strategies using parenteral acetaminophen as an adjunct have become increasingly popular as there are notable opioid-sparing effects demonstrated in surgical and anesthesia literature with minimal side effects and a low risk/benefit ratio.^3^,^4^,^9^–^16^ Intravenous acetaminophen has a diverse and broad compatibility with other agents, making it a successful adjunct to other agents, additional NSAIDS, and opioids.^14^ It also synergistically has been shown to increase analgesic affect in multimodal analgesia.^14^,^17^

Minimal literature is present regarding the opioid-sparing effects of parenteral acetaminophen outside of peri-operative settings.^12^ To our knowledge, only one study exists in the emergency medicine literature investigating the use of parenteral acetaminophen. Bektas et. al. compared 1,000 mg IV paracetamol (European name for acetaminophen) to morphine (0.1mg/kg) and placebo for the treatment of renal colic in the ED. Mean pain reduction and requirement of rescue analgesia was similar to morphine, with a noted trend in superiority in early pain assessment at 15 minutes.^5^

A recently published American Headache Society evidence assessment of migraine pharmacotherapies cited Level A evidence by Lipton et. al. demonstrating the efficacy of 1,000 mg of oral acetaminophen vs. placebo in treatment of acute migraine with regard to pain relief, functional disability, phonophobia and photophobia, though the study population was limited to those with minimal nausea and need for bed rest.^18^ This is a select population of patients that is perhaps less likely to present to the ED for treatment, though the documented efficacy of acetaminophen is quite profound. Additionally, patients presenting to the ED with severe headache often suffer from associated nausea and vomiting,^19^ further strengthening the potential application of parenteral acetaminophen where administration of oral formulation may not be possible. A pharmaceutical-sponsored study of OFIRMEV® (acetaminophen 1,000 mg/100 ml Cadence Pharmaceuticals) demonstrated peak IV acetaminophen plasma and cerebrospinal fluid concentrations were higher than oral or rectal acetaminophen.^19^.^20^ Additionally, IV acetaminophen does not undergo first-pass metabolism in the liver, reducing hepatic exposure to acetaminophen and thus diminishing the potential for hepatic injury.^16^,^19^,^21^

The use of IV acetaminophen as primary therapy for headaches would decrease the pitfalls of using primary NSAIDS such as ketorolac or ibuprofen in cases such as possible headache associated with intracranial hemorrhage where there is a platelet aggregation inhibition,^22^ potentially worsening clinical outcomes. Single doses of OFIRMEV® up to 3,000 mg and repeated doses of 1,000 mg every six hours for 48 hours have not been shown to cause a significant effect on platelet aggregation nor have any immediate or delayed effects on small vessels.^19^

Reviewing data findings, we obtained various pain scores in 30-minute intervals, of which only the first three pain scores (after the initial assessment) for a total of 90 minutes post-medication administration were considered. Pain scores were reported on a 1–10 VAS because of previously established integration with the her, thus enhancing data collection and ease of nursing-documented pain assessments essential to the study. Bijur et. al. reported decrease of pain by at least 1.4 as significant when investigating the VAS for pain reporting.^23^ We therefore considered a decrease in pain score reporting of ≥ 2 a “clinically significant” reduction.

The mean age of participants was 31 in males and 38 in females. This is consistent with reported headache-sufferer demographics according to the American Headache Society.^1^,^7^ A significantly higher portion of women (70) when compared to men (20) were noted as participants in the study. This demographic trend is consistent with data published by the Agency for Healthcare Research and Quality, which state that women typically outnumber men 3:1 in terms of presenting to EDs seeking treatment for acute headaches.^1^

The definition of rescue medications administered in this study included opioids, additional NSAIDS, steroids, orphenadrine, ergotamines, triptans or additional dopamine agonists. LOS was extrapolated from the time to disposition from first provider contact entered electronically per the EHR and included in both groups additional rescue medications and additional reassessment times. The time to disposition for either arm was very similar, which was an unexpected finding given the trend towards superior pain reduction in the acetaminophen group at 90 minutes. This may be attributed to the small size of our study, thus relatively small number of patients requiring rescue analgesia. In theory, those with improved pain reduction should require less rescue medications and would be suitable for discharge sooner. The maximum LOS for either treatment arm was 361 minutes in which the particular patient required significantly longer assessment due to refractory presentation. When compared to the additional subjects this was an outlier and did not greatly alter the data significance.

During enrollment, several physicians cited concern with excluding analgesic medications such as ketorolac from initial treatment. Several studies have demonstrated the superiority of combination metoclopramide plus diphenhydramine over NSAIDS,^24^,^25^ Regarding the efficacy of dopamine antagonist therapies for treatment of cephalgia, studies suggest a superiority of prochlorperazine to metoclopramide,^26^,^27^ though Friedman et. al. did not achieve statistical significance between treatment arms as opposed to prior studies.^27^ Diphenhydramine was administered to all patients due to the significantly reduced akathistic response with prophylactic administration.^28^ We believed the initial treatment regimen would be a reasonable and efficacious baseline regimen despite patients randomized to the placebo group not being given an NSAID medication upon initiation of treatment.

## LIMITATIONS

We identified some limitations during trial completion. Our intention was to enroll a consecutive series of eligible patients, but this relied on both patient and physician participation and consent to trial participation, which were both factors not within controlled limits of the study. Based on the projected sample size to achieve appropriate power, a sample size of (n=100) was deemed optimal; due to exclusion criteria and other factors as noted before, a sample size of (n=90) was ultimately available for analysis. While the ultimate study population was smaller than initially intended, we observed a greater outcome effect than anticipated such that statistical significance was still achieved, though this did require 90 minutes until a statistically significant difference was achieved. Individual emergency medicine providers were encouraged to enroll all eligible patients according to the study protocol; however, data displayed non-consecutive enrollment. We speculate this may have been due to some provider reluctance to participate in the study or patient refusal, preventing consecutive series enrollment. The degree of subject refusal was not recorded during the enrollment period for further reflection. At time of patient enrollment, treatment was initiated in both arms with initial administration of prochlorperazine and diphenhydramine within several minutes. In either arm, the “study drug” required the blinded product to be sent from pharmacy to the ED, resulting in subsequent administration to the initial medications as noted above. The level of effect of this on study outcomes is difficult to determine, since as noted in the placebo group the time to significant pain score decrease was slower than the acetaminophen group and pain score decrease more profound in the acetaminophen group, although both arms had delayed “study drug” administration by up to 15 minutes post initial medications. To maximize our sample size and decrease exclusion burden, we did not target a specific subset of headache populations. Total patient LOS was defined as arrival to the ED and time to disposition. The beginning of LOS was not recorded as initial provider assessment and study enrollment, which is certainly a confounding variable. The observed difference in LOS between the two study groups was not substantially different, and it is unclear if further analysis in this regard would have significantly changed the reported LOS between the groups.

It would be beneficial to delineate in a larger trial if the observed benefit of IV acetaminophen is specific to certain headache conditions. Going forward, it would be worthwhile to study a head-to-head comparison of IV acetaminophen alone with a standard NSAID or opioid therapy to ascertain if similar efficacy exists in treatment of cephalgia as it was reported in treatment of renal colic by Bektas et. al.^5^

Results may further support evidence suggesting that avoidance of opioids in treatment of headache presentations is wise. It is also worthwhile to note that a cost analysis was not performed in this trial, and all medications were provided without cost to patients involved in the trial. This is important as OFIRMEV® as currently available in clinical practice does carry moderate increase in patient cost compared to therapies that have been traditionally used, and may represent a different or increased billing charge toward the patient. Further investigation is required to weight financial burden versus therapeutic effect.

## CONCLUSION

IV acetaminophen when used with prochlorperazine and diphenhydramine to treat acute headaches in the ED resulted in statistically significant pain reduction compared with prochlorperazine and diphenhydramine alone as measured by both threshold of lowering VAS pain score by at least two points (NNT = 4) and overall decline in VAS pain score. Further study is required to validate these results.

## Figures and Tables

**Figure 3:**
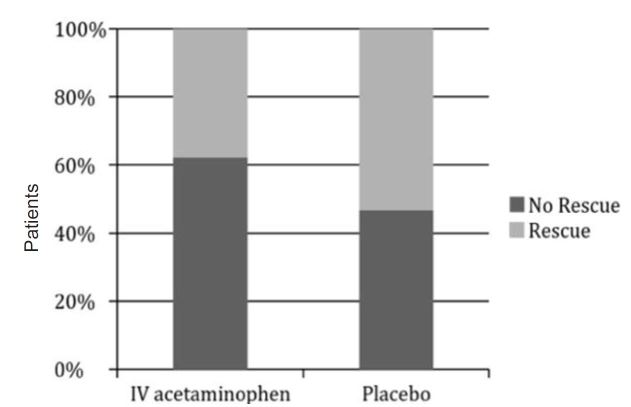
Comparative percentages of those requiring rescue analgesia in both IV (intravenous) acetaminophen and placebo.

**Table:** Individual race, as per hospital federal reporting regulations, of participants in a study of the effectiveness of adding parenteral acetaminophen in treatment of acute headache.

Demographics	n
Black or African Americans	46
White, Hispanic, or Caucasian	43
Asian/Pacific Islander	0
American Indian or Alaskan Native	1
